# Advances in Regenerative Dentistry: From Biological Mechanisms to Clinical Translation

**DOI:** 10.3390/life16091579

**Published:** 2026-09-21

**Authors:** Mariela Ramirez-Velasquez, Gabriela Del Cisne Valarezo-Chicaiza, Gabriela Cristina Astudillo-Rubio, Stephany Paladines-Calle

**Affiliations:** Grupo de Investigación e Innovación en Salud Oral Integral (GIISO), Carrera de Odontología, Universidad Politécnica Salesiana, Cuenca 010101, Ecuador; gvalarezo@ups.edu.ec (G.D.C.V.-C.); gastudillo@ups.edu.ec (G.C.A.-R.); spaladines@ups.edu.ec (S.P.-C.)

**Keywords:** regenerative dentistry, oral tissue regeneration, tissue engineering, dental stem cells, bioactive biomaterials, cell-free therapy, regenerative medicine

## Abstract

Advances in cell biology, biomaterials, and tissue engineering have expanded the possibilities of regenerative dentistry, promoting a shift from tissue replacement toward biologically driven strategies aimed at restoring tissue structure and function. This review examines advances reported between 2020 and 2025, considering their biological basis, current evidence, and level of clinical translation. Major developments include regenerative endodontics, periodontal regeneration, guided tissue and bone regeneration, platelet concentrates, bioactive biomaterials, dental stem cells and cell-free therapies, biomimetic enamel regeneration, and organoid and whole-tooth bioengineering. Although some approaches are already supported by human evidence and clinical application, others remain predominantly experimental. Importantly, favorable healing or tissue formation does not necessarily represent complete functional regeneration of the native tissue. Greater standardization, long-term clinical evidence, and reproducible therapeutic protocols are therefore needed to bridge the gap between experimental advances and predictable clinical outcomes.

## 1. Introduction

Regenerative dentistry has moved beyond the concept of replacing damaged oral tissues toward strategies that seek to restore their biological structure and function. This transition has been supported by a better understanding of the cellular and molecular mechanisms involved in tooth development, tissue homeostasis, and repair, particularly epithelial–mesenchymal interactions, stem and progenitor cell behavior, extracellular matrix signaling, and the molecular pathways that regulate cell differentiation and tissue organization [1,2,3,4]. These biological principles now provide the basis for regenerative approaches targeting the pulp–dentin complex, periodontium, cementum, alveolar bone, and other dental tissues.

Between 2020 and 2025, this field has expanded considerably, with progress extending beyond conventional stem cell and scaffold-based tissue engineering. Regenerative endodontic procedures, periodontal and guided tissue regeneration, guided bone regeneration, platelet-derived products, and bioactive biomaterials have continued to evolve, while cell-free strategies based on secretomes and extracellular vesicles have gained increasing attention [5,6,7]. At the same time, biomimetic approaches for enamel remineralization and emerging technologies such as dental organoids, biofabrication, nanotechnology, and whole-tooth bioengineering have broadened the possibilities of dental tissue regeneration [8,9,10]. Importantly, these approaches are not progressing at the same pace: some are already used clinically or supported by human studies, whereas others remain largely at the experimental or preclinical stage [6,7].

Several recent reviews have examined regenerative dentistry from different perspectives. Some have focused primarily on stem cells and their applications in dental, periodontal, and bone tissue regeneration, whereas others have emphasized biomaterials, scaffolds, nanomaterials, or specific clinical applications [6,7,8,9]. For example, the systematic review by Inchingolo et al. [7] centered on stem-cell-based strategies and tissue engineering, particularly for pulp, periodontal, and bone regeneration, while Thalakiriyawa and Dissanayaka [6] provided a concise overview of emerging clinical strategies with particular attention to dental stem cells, regenerative endodontics, periodontal regeneration, and bone regeneration. Other reviews have addressed advanced biomaterials and oral tissue engineering or specifically examined nanomaterials as regenerative agents [8,9]. Despite these contributions, the literature remains fragmented across biological mechanisms, individual technologies, and specific dental applications. A broader synthesis that follows recent advances from their biological basis through regenerative strategy and experimental evidence to their current clinical relevance is therefore still needed.

At the biological level, oral tissue regeneration depends on the coordinated response of resident specialized cells together with stem and progenitor cell populations. Odontoblasts, cementoblasts, periodontal ligament fibroblasts, epithelial cells, and other tissue-specific populations contribute to tissue maintenance and repair, while stem and progenitor cells provide a source of cells capable of proliferating and differentiating in response to regenerative signals [11,12]. These processes are strongly influenced by the extracellular matrix, which not only provides structural support but also regulates cell adhesion, migration, proliferation, and differentiation through interactions with growth factors, cytokines, and cell-surface receptors [13,14]. In this way, the regenerative response depends not on isolated cell populations, but on their interaction within a dynamic tissue microenvironment.

This microenvironment is further regulated by molecular signaling and immune activity. Pathways such as bone morphogenetic proteins, fibroblast growth factors, Wnt, transforming growth factor-β, and Sonic Hedgehog contribute to the control of stem and progenitor cell behavior, odontogenic and osteogenic differentiation, angiogenesis, and extracellular matrix formation [15,16]. At the same time, inflammatory and immune responses can either support or interfere with tissue repair. Controlled inflammation may promote cell recruitment and the release of signaling molecules, whereas persistent or dysregulated inflammation can impair regeneration and favor extracellular matrix degradation, particularly in pulpal and periodontal tissues [17,18].

Successful regeneration therefore requires a balanced interaction among cell recruitment, proliferation, differentiation, extracellular matrix remodeling, immune regulation, vascular support, and tissue-specific signaling [19,20]. These mechanisms provide the biological foundation for current regenerative strategies and help explain why therapeutic outcomes vary according to the target tissue, local microenvironment, biomaterial, and stage of clinical translation. Their integration is essential for understanding how recent regenerative approaches have progressed from basic biological concepts toward more clinically oriented applications.

Accordingly, this narrative review critically examines major developments in regenerative dentistry reported between 2020 and 2025, linking the cellular and molecular mechanisms that support tissue repair with the regenerative strategies derived from them and the evidence supporting their translation into clinical practice. Rather than providing an exhaustive description of basic cell biology, the review focuses on developments that have meaningfully advanced the field, including regenerative endodontics and pulp–dentin regeneration; periodontal and cementum–periodontal ligament regeneration; guided tissue and bone regeneration; platelet concentrates and autologous regenerative products; alveolar bone regeneration and bioactive biomaterials; dental stem cells and cell-free strategies; biomimetic enamel remineralization and regeneration; and dental organoids, biofabrication, and whole-tooth bioengineering. Particular attention is given to the distinction between preclinical and human evidence, the current clinical relevance of each approach, and the biological, technical, regulatory, and translational barriers that still limit their broader application.

## 2. Materials and Methods

### 2.1. Review Design and Literature Search

This narrative review was designed to provide a critical and translational overview of recent developments in regenerative dentistry. The literature search focused primarily on studies published between 2020 and 2025, as this period was selected to capture the most recent advances in regenerative strategies and their progression toward clinical application. Earlier studies were considered only when they provided essential biological or conceptual background that could not be adequately supported by more recent literature.

A literature search was conducted in PubMed/MEDLINE, Scopus, and Web of Science. Search terms were adapted to each database and included combinations of terms related to *regenerative dentistry*, *dental tissue regeneration*, *regenerative endodontics*, *pulp–dentin regeneration*, *periodontal regeneration*, *cementum regeneration*, *periodontal ligament*, *guided tissue regeneration*, *guided bone regeneration*, *platelet concentrates*, *PRP*, *PRF*, *alveolar bone regeneration*, *bioactive biomaterials*, *dental stem cells*, *extracellular vesicles*, *exosomes*, *cell-free therapy*, *enamel remineralization*, *biomimetic enamel regeneration*, *dental organoids*, *3D bioprinting*, *tooth germ engineering*, and *whole-tooth regeneration.* Additional relevant studies were identified by screening the reference lists of selected articles.

### 2.2. Selection of Major Advances

Rather than attempting to provide an exhaustive description of all developments in regenerative dentistry, the review focused on advances considered to have meaningfully contributed to the field during the 2020–2025 period. A development was considered relevant when it met one or more of the following criteria: (1) introduced or substantially improved a regenerative strategy; (2) provided new evidence supporting the biological mechanisms underlying tissue regeneration; (3) demonstrated progression from in vitro research toward animal or human investigation; (4) showed clinically relevant outcomes or increasing potential for clinical translation; or (5) addressed an important limitation of previously available regenerative approaches.

The selected advances were subsequently organized into eight major areas according to the primary tissue or regenerative target, the biological or technological strategy involved, and their current level of development within regenerative dentistry. This grouping was intended to distinguish established clinically oriented approaches from emerging regenerative strategies while avoiding overlap between closely related technologies. The resulting areas were regenerative endodontics and pulp–dentin regeneration; periodontal regeneration and cementum–periodontal ligament engineering; guided tissue and guided bone regeneration; autologous platelet concentrates; alveolar bone regeneration and bioactive biomaterials; dental stem cells and cell-free strategies; biomimetic enamel regeneration; and biofabrication, organoids, and whole-tooth regeneration. These categories were selected because they represented the main recurring and clinically or biologically relevant lines of advancement identified in the literature published during 2020–2025.

### 2.3. Evidence Synthesis and Translational Assessment

Evidence from in vitro studies, animal models, clinical studies, randomized clinical trials, systematic reviews, and meta-analyses was considered when relevant to the purpose of each section. Greater emphasis was placed on primary studies to support specific mechanistic and therapeutic findings, while systematic reviews and meta-analyses were used to provide a broader perspective on the consistency and clinical relevance of the available evidence.

The selected evidence was synthesized narratively using a common translational framework that considered the biological basis of each approach, the regenerative strategy involved, the available preclinical and human evidence, and its current clinical relevance. Particular attention was given to distinguishing promising experimental findings from evidence supporting clinical application. Accordingly, regenerative approaches were discussed according to their current stage of development, ranging from predominantly experimental or preclinical strategies to those supported by emerging or established clinical evidence. This framework was also used to identify the main limitations and evidence gaps that continue to affect clinical translation.

## 3. Biological Foundations of Oral Tissue Regeneration

### 3.1. Cellular and Molecular Basis of Oral Tissue Regeneration

Oral tissues contain specialized cell populations whose coordinated activity supports tissue development, homeostasis, and repair. Ameloblasts are responsible for enamel formation and also contribute to regenerative processes mediated by enamel matrix proteins [10,21], whereas odontoblasts are essential for dentin formation and maintenance [22,23]. Cementoblasts contribute to cementum formation and periodontal regeneration [11,24,25,26], while periodontal ligament fibroblasts regulate collagen synthesis and remodeling and play an important role in periodontal healing and structural maintenance [12,27,28]. Oral epithelial cells also contribute to tissue protection, maintenance of epithelial integrity, and regenerative responses following injury [29].

From a regenerative perspective, however, tissue repair depends not only on the presence of these individual cell populations but also on their interaction with the surrounding microenvironment. The extracellular matrix provides structural support while regulating cell adhesion, migration, proliferation, and differentiation through interactions with growth factors, cytokines, and cell-surface receptors [13,14]. These interactions influence the behavior of dental stem and progenitor cells and contribute to the organization of newly formed tissues.

Cellular metabolism and protein synthesis also support regenerative activity by providing the energy and extracellular matrix components required for tissue remodeling. In highly secretory cells such as odontoblasts and ameloblasts, protein synthesis and processing are particularly important for the production of matrix components involved in dentin and enamel formation [30,31,32,33]. Similarly, mitochondrial activity contributes to cellular metabolism and signaling during tissue repair and remodeling [34,35]. Rather than acting independently, these cellular processes form part of an integrated biological environment that determines how oral tissues respond to injury and regenerative stimuli.

### 3.2. Cell Signaling and the Regenerative Microenvironment

Regeneration of oral tissues requires continuous communication among resident cells, stem and progenitor cells, immune cells, and the extracellular matrix. This communication occurs through direct cell–cell interactions, extracellular matrix-mediated signaling, and soluble mediators such as cytokines and growth factors [13,14,36,37,38]. Together, these mechanisms regulate cell migration, proliferation, differentiation, extracellular matrix remodeling, and tissue organization.

Several signaling pathways involved in dental development are also reactivated or modulated during tissue repair. Bone morphogenetic proteins (BMPs), fibroblast growth factors (FGFs), Wnt, transforming growth factor-β (TGF-β), and Sonic Hedgehog (SHH) signaling contribute to the regulation of stem and progenitor cell behavior, odontogenic and osteogenic differentiation, angiogenesis, and extracellular matrix formation [15,16,39]. Their coordinated activity illustrates an important principle of regenerative dentistry: many therapeutic strategies seek to reproduce or modulate biological signals that normally guide tissue development and healing.

The regenerative microenvironment is also shaped by inflammatory and immune responses. Controlled inflammation can support tissue repair by recruiting cells and releasing signaling molecules, whereas persistent or dysregulated inflammation may impair regeneration and promote extracellular matrix degradation and tissue destruction [17,18]. This distinction is particularly relevant in periodontal and pulpal tissues, where the local inflammatory environment can influence the response to regenerative procedures and biomaterials [17,18]. Understanding these interactions therefore provides the biological basis for regenerative strategies designed not only to replace damaged tissue but also to create a microenvironment favorable to healing and functional restoration.

### 3.3. Cellular Proliferation, Differentiation, and Regenerative Response

Successful tissue regeneration requires a controlled balance between cell proliferation, differentiation, and survival. In oral tissues, these processes contribute to the renewal and repair of the dental pulp, periodontium, oral epithelium, and supporting tissues following injury [19,20]. Stem and progenitor cells must proliferate sufficiently to provide an adequate cellular population and subsequently differentiate into specialized phenotypes capable of rebuilding damaged tissue.

Cell-cycle regulation is therefore relevant to regenerative dentistry, although its importance lies primarily in the control of regenerative cell behavior rather than in the individual phases of the cycle. Cyclins, cyclin-dependent kinases, and checkpoint proteins regulate cellular proliferation and preserve genomic stability during cell expansion [40,41,42]. This balance is particularly important when stem cells are expanded or stimulated for regenerative purposes since uncontrolled proliferation or impaired checkpoint regulation may compromise both tissue organization and biological safety.

**Figure 1:**
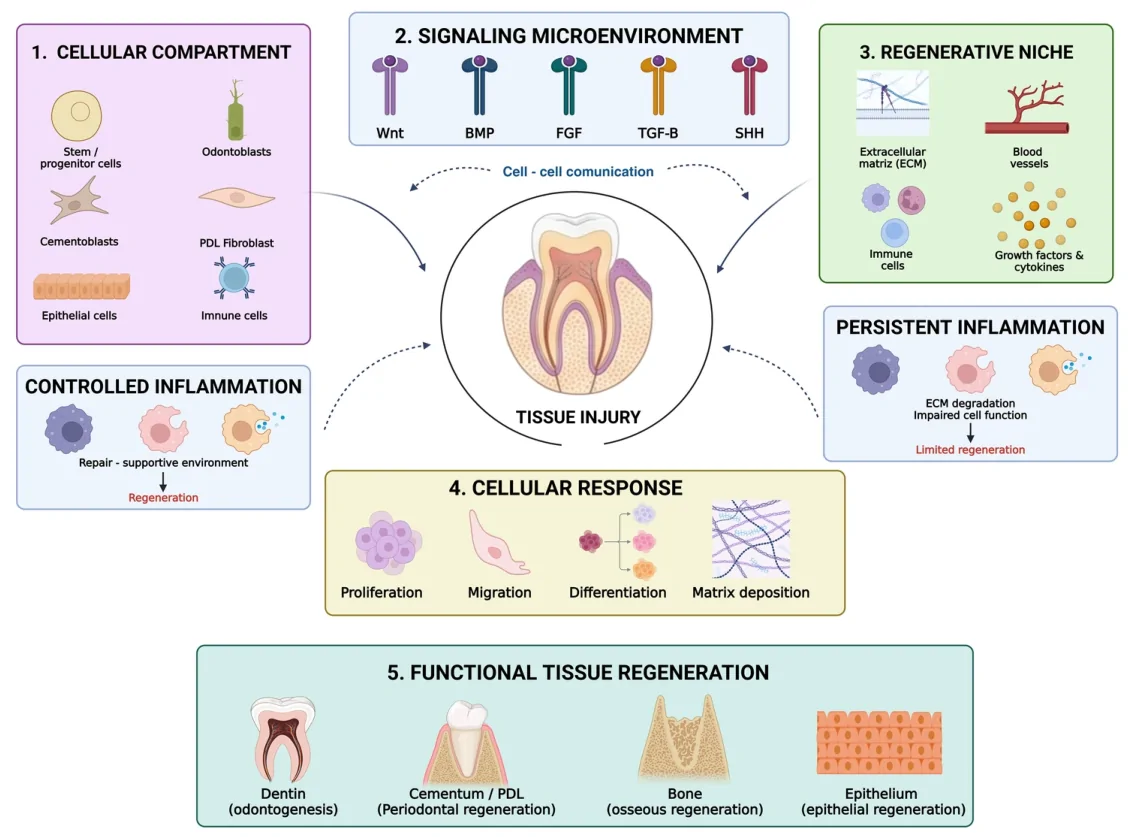
Integrated cellular and molecular mechanisms underlying oral tissue regeneration. Oral tissue regeneration results from the coordinated interaction of specialized and progenitor cells, signaling pathways, extracellular matrix components, vascular and immune factors, and controlled cellular responses. These interactions regulate proliferation, migration, differentiation, and matrix deposition, while the inflammatory microenvironment may either support or limit the regenerative process. Together, these mechanisms contribute to the functional regeneration of dentin, periodontal tissues, bone, and oral epithelium. Created in BioRender. Valarezo, G. (2026). https://BioRender.com/dwzveaz.

The regenerative response ultimately depends on the integration of these cellular events with extracellular signals, vascular support, immune regulation, and the local extracellular matrix. For this reason, current regenerative approaches increasingly aim to modulate the cellular microenvironment rather than stimulate proliferation alone. As summarized in Figure 1, these interconnected cellular and molecular mechanisms provide the biological foundation for tissue regeneration and help explain why regenerative outcomes may vary according to the target tissue, local microenvironment, biomaterial, and stage of clinical translation.

## 4. Recent Advances in Regenerative Dentistry

**Figure 2:**
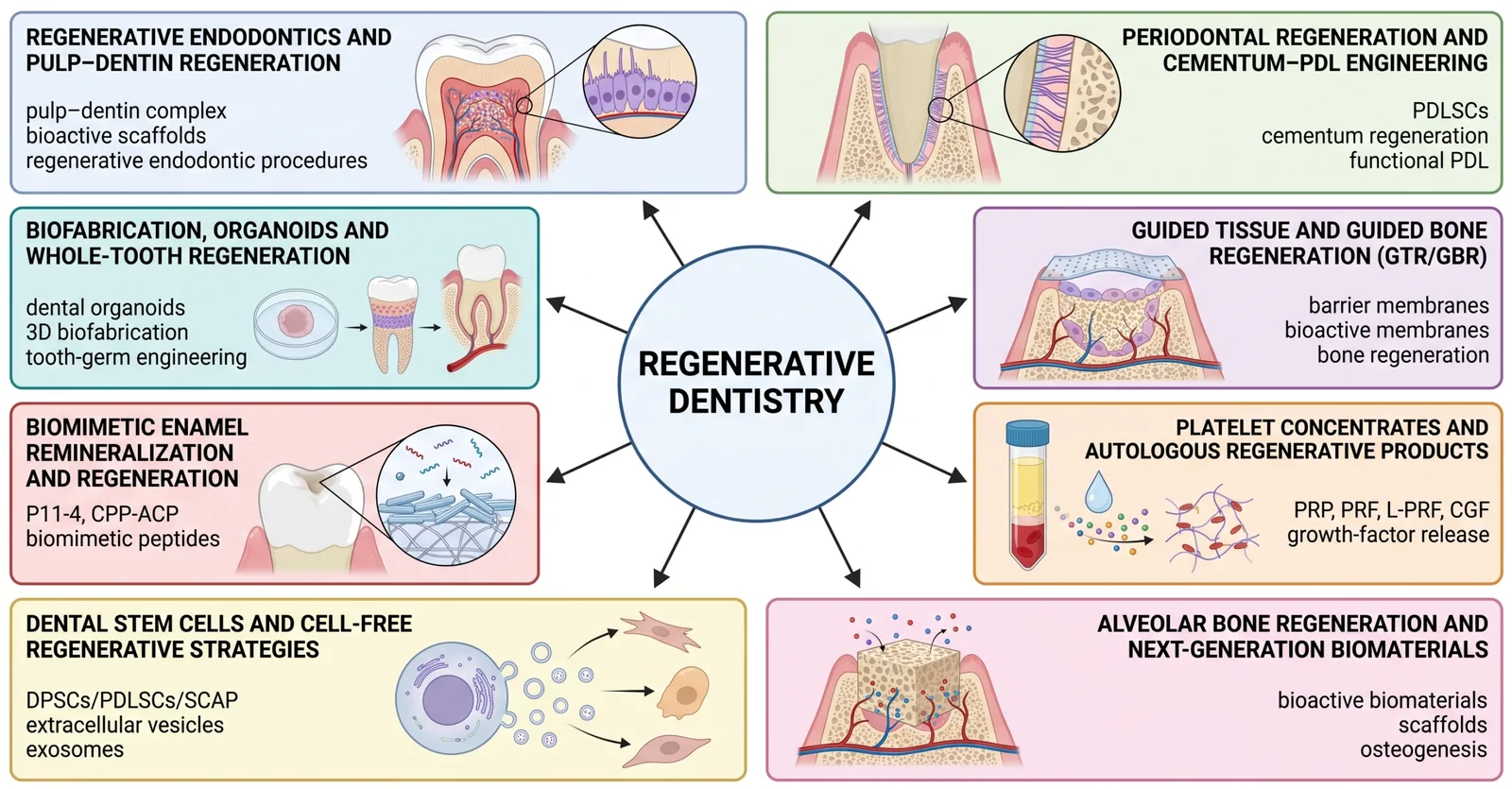
Major advances in regenerative dentistry. Schematic overview of the eight regenerative areas addressed in this review, including regenerative endodontics and pulp–dentin regeneration, periodontal regeneration and cementum–periodontal ligament engineering, guided tissue and guided bone regeneration (GTR/GBR), platelet concentrates and autologous regenerative products, alveolar bone regeneration and bioactive biomaterials, dental stem cells and cell-free regenerative strategies, biomimetic enamel remineralization and regeneration, and organoid- and whole-tooth-based bioengineering approaches. Created in BioRender. Valarezo, G. (2026). https://BioRender.com/vpsopd9.

Recent advances in regenerative dentistry encompass multiple biological, biomaterial-based, and bioengineering approaches directed toward the repair or regeneration of oral tissues. For the purposes of this review, these developments were organized into eight major regenerative areas, as summarized in Figure 2.

### 4.1. Regenerative Endodontics and Pulp–Dentin Regeneration

Regenerative endodontics has become one of the most clinically developed areas of regenerative dentistry, particularly for immature permanent teeth with pulp necrosis. Unlike conventional endodontic treatment, regenerative approaches aim not only to eliminate infection but also to restore biological activity within the pulp–dentin complex, potentially supporting continued root development, thickening of dentinal walls, and apical maturation. Current regenerative endodontic procedures (REPs) combine canal disinfection, recruitment of endogenous cells, a biological or engineered scaffold, and signaling molecules that support tissue ingrowth and repair [43,44]. Clinical evidence is strongest for immature permanent teeth, whereas outcomes in mature teeth remain less predictable [44,45].

Current strategies can broadly be divided into cell-free and cell-based approaches. Cell-free REPs recruit endogenous stem or progenitor cells into the disinfected canal, commonly through apically induced bleeding and formation of a blood clot or another biological scaffold. Cell-based approaches instead introduce exogenous cells, particularly dental pulp stem cells (DPSCs), which have odontogenic, proliferative, and neurovascular potential [43,46]. Cell homing is clinically attractive because it avoids ex vivo cell expansion and transplantation, whereas cell-based therapy may provide greater control over the regenerative cellular component and has shown potential for pulp-like tissue formation. Nevertheless, its clinical development remains limited [43,46,47].

A major biological challenge is that functional pulp regeneration requires more than vascularized connective tissue. Ideally, regenerated tissue should reproduce vascular and neural networks together with an organized odontoblast-like layer capable of depositing tubular dentin [47,48]. This is particularly difficult in narrow or mature canals, where restricted vascular access may compromise cell survival and tissue organization. Consequently, recent research has increasingly focused on controlling the regenerative microenvironment through biomimetic scaffolds, hydrogels, growth factors, and developmental signaling pathways. Wnt/β-catenin signaling, for example, has attracted attention because of its involvement in odontogenic differentiation and tertiary dentin formation [49]. However, achieving coordinated vascularization, innervation, and organized dentin formation remains a major challenge [47,48,49].

Importantly, favorable clinical outcomes after REPs should not automatically be interpreted as true histological regeneration. Current procedures can promote resolution of symptoms, periapical healing, and continued root maturation, but histological studies indicate that the tissue formed after cell-free procedures may predominantly resemble connective, bone-like, or cementum-like tissue rather than a fully organized pulp–dentin complex [43,50]. Current cell-free REPs may therefore be more accurately interpreted as biologically guided repair despite their clear clinical value [43]. This distinction is essential because radiographic root development or recovery of sensibility does not necessarily demonstrate restoration of native pulp architecture.

From a translational perspective, cell-free REPs are already used clinically, particularly in necrotic immature permanent teeth, whereas cell-based pulp regeneration remains closer to the clinical-trial stage [43,44,46,47]. Outcomes continue to depend on infection control, apical anatomy, regenerative cell availability, scaffold characteristics, vascular supply, and protocol variability [43,44,51]. Thus, the central challenge is shifting from simply obtaining tissue within the root canal toward reproducing the structural and biological functions of the native pulp–dentin complex. Future progress will depend on better control of vascularization, innervation, odontoblastic differentiation, and tissue organization, together with standardized protocols and stronger long-term human evidence [43,47,48]. Overall, the major advance during 2020–2025 was the progression of regenerative endodontics from primarily revascularization-based procedures toward more biologically guided strategies aimed at achieving a more organized and functional pulp–dentin complex.

### 4.2. Periodontal Regeneration and Cementum–Periodontal Ligament Engineering

Periodontal regeneration is particularly challenging because it requires the coordinated reconstruction of several tissues rather than the formation of a single structure. True regeneration involves new cementum, a functionally oriented periodontal ligament (PDL) with fibers inserted into both cementum and alveolar bone, and restoration of supporting bone [52,53]. Although current regenerative procedures can improve clinical outcomes, their predictability remains influenced by defect morphology, local conditions, and case selection, and complete restoration of the periodontal attachment apparatus is still difficult to achieve consistently [52].

For this reason, recent tissue-engineering strategies have focused on reproducing the cementum–PDL–bone complex as an integrated unit. The PDL contains heterogeneous cell populations, including mesenchymal stem/progenitor cells capable of contributing to periodontal repair [53,54]. Human periodontal ligament stem cells (hPDLSCs) have shown osteogenic, fibrogenic, and cementogenic differentiation, supporting their potential to regenerate several components of the periodontal complex rather than bone alone [54]. At the same time, signaling pathways such as Wnt/β-catenin are being investigated as biological targets to modulate cementum formation and regenerative cell behavior [55,56].

Biomaterial design has also evolved toward greater spatial control of regeneration. Patterned, compartmentalized, and multiphasic scaffolds are intended to provide different microenvironments for cementum, PDL, and bone formation, while cell-sheet approaches aim to preserve cell–cell interactions and extracellular matrix organization [57,58,59]. Preclinical studies illustrate the potential of this strategy: three-dimensional bone–ligament constructs have generated PDL-like fibers connecting the root surface with alveolar bone, supporting the feasibility of reconstructing periodontal interfaces rather than producing isolated mineralized tissue [59].

Another emerging direction is the modulation of the regenerative microenvironment. Experimental studies have explored bioactive agents capable of enhancing the osteogenic and cementogenic activity of hPDLSCs; for example, metformin has been associated with improved cell proliferation, migration, and differentiation, as well as increased bone and cementum formation in animal periodontal defects [60]. More broadly, current research is considering the influence of inflammation, microbial products, metabolic signals, epigenetic regulation, and immune–stem cell interactions on cementoblast differentiation and tissue repair [55,61]. These findings reinforce the idea that periodontal regeneration depends not only on delivering cells or scaffolds, but also on creating a local environment that supports their survival, organization, and integration.

Despite these advances, a substantial translation gap remains. Animal models generally show more favorable cementum–PDL regeneration than human studies, and species-related differences in cementogenesis and periodontal attachment limit direct extrapolation of preclinical findings [52,62]. Therefore, while stem-cell therapies, signaling modulation, cell sheets, and advanced scaffolds have broadened the regenerative possibilities of the field, many remain predominantly experimental [52,57,62]. The main challenge is no longer simply to demonstrate new bone or cementum formation, but to achieve predictable fiber orientation, stable tissue integration, and long-term functional restoration of the periodontal attachment apparatus in humans. Overall, the major advance during 2020–2025 was the development of more integrated regenerative strategies aimed at reconstructing the cementum–PDL–bone complex as a functional unit, rather than regenerating its individual components separately.

### 4.3. Guided Tissue and Guided Bone Regeneration (GTR/GBR)

Guided tissue regeneration (GTR) and guided bone regeneration (GBR) are among the most established regenerative approaches in dentistry. Both rely on the use of barrier membranes to exclude rapidly proliferating epithelial and connective tissue cells and maintain a protected space for cells involved in periodontal or bone regeneration. Their therapeutic goals, however, differ: GTR aims to restore the periodontal attachment apparatus, including cementum, periodontal ligament, and alveolar bone, whereas GBR is primarily used for alveolar bone reconstruction and augmentation [63,64,65,66,67]. Successful regeneration therefore depends not only on cell exclusion but also on adequate space maintenance, vascularization, and membrane stability during healing.

Membrane properties are critical to these outcomes. An ideal barrier should provide biocompatibility, cell occlusivity, tissue integration, sufficient mechanical stability, appropriate porosity, and a degradation profile compatible with the rate of tissue formation [63,64,67,68]. Non-resorbable membranes, including expanded polytetrafluoroethylene and titanium-reinforced systems, provide effective space maintenance but require surgical removal and may increase treatment burden. Resorbable membranes, particularly collagen-based materials, avoid a second surgical procedure and offer favorable biocompatibility, although rapid degradation and limited mechanical stability can reduce their effectiveness in larger or poorly contained defects [63,66,67,68].

Recent advances have therefore focused less on the basic principle of guided regeneration and more on transforming membranes from passive barriers into bioactive regenerative interfaces. Natural and synthetic polymers, composite systems, inorganic or nanoscale components, and incorporated therapeutic agents are being investigated to improve mechanical performance while promoting cellular adhesion, osteogenic activity, and antimicrobial effects [64,65,66,67]. Manufacturing technologies such as electrospinning can generate fibrous structures resembling the extracellular matrix, while additive manufacturing and three-dimensional printing provide greater control over membrane architecture, porosity, and geometry [65,67].

This evolution is particularly evident in asymmetric and functionally graded membranes, in which different regions are designed to perform specific biological functions. A dense surface can restrict soft-tissue invasion, whereas a porous or bioactive surface facing the defect can favor cell attachment, vascular ingrowth, and new tissue formation [63,68]. Chitosan-based composites and other multifunctional systems have also been explored to combine barrier properties with osteoconductive, antimicrobial, or biologically active functions [68]. These designs illustrate the current shift toward membranes that actively influence the regenerative microenvironment rather than simply separating tissues.

From a translational perspective, however, a clear distinction remains between established and emerging technologies. Conventional collagen and non-resorbable membranes already have substantial clinical applicability, whereas electrospun constructs, nanocomposites, asymmetric and functionally graded membranes, and advanced three-dimensional designs remain supported mainly by experimental or early translational evidence [63,64,65,66,67,68]. Thus, the major advance in GTR/GBR during 2020–2025 has been the development of increasingly bioactive and structurally tailored membranes, but their improved biological performance in preclinical models cannot yet be assumed to provide superior clinical regeneration. Future progress will depend on demonstrating that these technological improvements produce reproducible long-term benefits compared with established membrane systems. Between 2020 and 2025, GTR/GBR advanced toward multifunctional membrane systems that combine barrier function with greater biological activity and more precise control of the regenerative environment.

### 4.4. Platelet Concentrates and Autologous Regenerative Products

Autologous platelet concentrates (APCs) provide a clinically accessible source of fibrin, platelets, leukocytes, and bioactive mediators involved in wound healing and tissue repair. Their development has progressed from platelet-rich plasma (PRP) toward fibrin-based preparations such as platelet-rich fibrin (PRF) and leukocyte- and platelet-rich fibrin (L-PRF), which differ in cellular composition, fibrin architecture, preparation protocols, and growth-factor release kinetics [69,70,71]. This evolution has shifted their role from simple reservoirs of concentrated growth factors toward autologous matrices capable of providing temporary structural support and a biologically active environment for healing.

PRF-based products have consequently been investigated across periodontal and oral regenerative procedures, including intrabony and furcation defects, extraction sockets, ridge preservation, sinus augmentation, guided bone regeneration, and peri-implant healing [70]. Their three-dimensional fibrin network can retain platelets, leukocytes, and signaling molecules and provide a more sustained release profile than PRP [69,71]. However, these biological advantages do not necessarily translate into superior clinical outcomes in every indication. In implant therapy, for example, PRP has not shown a consistent additional benefit for implant stability or osseointegration, whereas L-PRF may improve some early healing parameters without clear evidence that these advantages persist over longer follow-up [72].

A similar pattern has been observed in endodontic applications. APCs used as adjuncts in endodontic surgery have been associated with improvements in postoperative symptoms and radiographic healing, but heterogeneity and risk of bias limit the strength of these conclusions [73]. More importantly, a 2025 systematic review and meta-analysis of 15 randomized clinical trials found no significant difference in overall clinical and radiographic success at 12 months when either PRP or PRF was compared with conventional blood-clot revascularization [74]. These findings suggest that platelet concentrates can provide useful biological scaffolds and may enhance particular aspects of healing, but their use should not be interpreted as inherently superior to established regenerative protocols.

A persistent limitation is the lack of standardization among APC preparations. Centrifugation conditions, tube characteristics, leukocyte and platelet content, fibrin architecture, and individual patient factors can alter the composition and biological behavior of the final product [69,71,72]. Recent developments have therefore focused on refining centrifugation protocols and designing PRF formulations with more reproducible cellular and fibrin characteristics [70]. Overall, APCs are already integrated into clinical regenerative dentistry, but their principal role appears to be as adjunctive autologous bioactive matrices rather than stand-alone regenerative therapies. Establishing which formulations provide clinically meaningful benefits for specific indications will require more standardized protocols and stronger comparative long-term evidence [70,72,73,74]. The most notable progress during this period was the refinement of fibrin-based platelet concentrates as autologous bioactive matrices and their increasing integration as adjuncts across a range of regenerative procedures.

**Figure 3:**
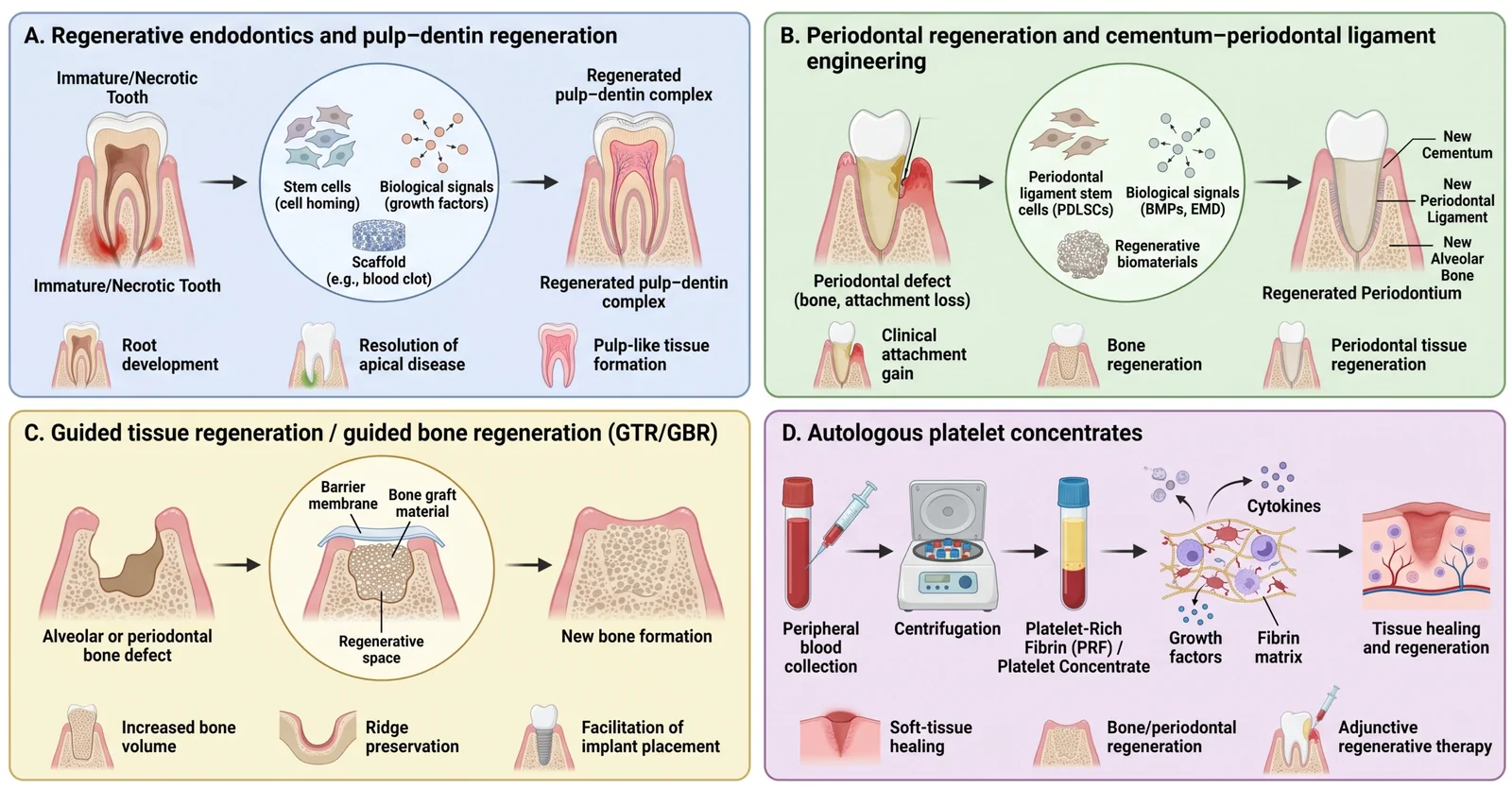
Established and clinically translated regenerative approaches in dentistry. Schematic representation of four major regenerative strategies with current clinical relevance: (**A**) regenerative endodontics and pulp–dentin regeneration; (**B**) periodontal regeneration and cementum–periodontal ligament engineering; (**C**) guided tissue regeneration and guided bone regeneration (GTR/GBR); and (**D**) autologous platelet concentrates. The figure illustrates the principal biological or therapeutic components involved in each approach and their main regenerative outcomes. Created in BioRender. Valarezo, G. (2026). https://BioRender.com/gkelahl.

Together, these approaches illustrate the spectrum of regenerative strategies that have achieved the greatest degree of clinical translation, ranging from biologically guided tissue repair to membrane-, biomaterial-, and autologous product-based interventions. Their main regenerative mechanisms, target tissues, and clinically relevant outcomes are summarized in Figure 3.

### 4.5. Alveolar Bone Regeneration and Next-Generation Biomaterials

Alveolar bone regeneration is essential for periodontal reconstruction and implant rehabilitation, particularly when bone loss compromises ridge dimensions or implant placement. Although autologous bone remains biologically advantageous, its availability and donor-site morbidity have encouraged the development of synthetic and bio-derived substitutes [75,76]. Among the most established alternatives are hydroxyapatite (HA), β-tricalcium phosphate (β-TCP), biphasic calcium phosphate (BCP), and bioactive glasses. Their regenerative performance depends not only on composition but also on porosity, surface characteristics, mechanical stability, and degradation kinetics, which influence vascularization, cellular infiltration, and bone remodeling [75,77].

Importantly, several of these materials have progressed beyond preclinical investigation. A 2025 systematic review of 11 comparative clinical studies reported satisfactory bone regeneration and implant stability with synthetic biomaterials, with bone preservation generally ranging from 85% to 95%. β-TCP and BCP showed favorable resorption and dimensional stability, while bioactive glass demonstrated good integration and remodeling. In several clinical settings, particularly socket preservation and ridge augmentation, their performance was comparable to xenografts [75]. These findings support the clinical relevance of synthetic substitutes, although the available evidence does not establish a single material as universally superior.

A major recent development has been the transition from materials that primarily provide osteoconductive support toward bioactive and bioinstructive platforms. Ion-releasing ceramics, functionalized scaffolds, hydrogels, bioactive coatings, and controlled-delivery systems are being designed to interact with the local microenvironment and influence osteogenesis, angiogenesis, inflammation, and tissue integration [76,78]. Three-dimensional printing, nanotechnology, and composite systems further allow greater control over scaffold architecture, porosity, surface properties, and adaptation to individual defects [76,78]. However, many of these advanced systems remain supported predominantly by experimental evidence, and greater technological complexity has not yet been shown to consistently provide superior clinical outcomes.

Bio-derived alternatives have also gained attention. Demineralized dentin matrix (DDM), obtained from autologous teeth, provides a collagen-rich extracellular matrix containing biologically active components and avoids the need for a second bone-harvesting site. A 2023 systematic review reported favorable biocompatibility and bone regenerative properties for autologous tooth-derived grafts [79]. Nevertheless, differences in processing methods and limited standardized comparative evidence still prevent these materials from being considered equivalent to established grafting approaches across all clinical indications.

Overall, alveolar bone regeneration is progressing from conventional defect filling toward materials designed to actively participate in the regenerative process. Established synthetic substitutes already provide clinically useful options, whereas bioinstructive scaffolds, advanced delivery systems, and personalized constructs represent a more recent translational stage [75,76,78]. No single biomaterial currently reproduces all the biological properties of autologous bone, and material selection therefore remains dependent on defect morphology, vascular supply, mechanical requirements, degradation behavior, and clinical indication. The next challenge is to determine whether the biological advantages demonstrated by increasingly sophisticated biomaterials translate into reproducible and clinically meaningful improvements over established regenerative strategies. The key progress achieved during 2020–2025 was the expansion of clinically established bone substitutes alongside the development of increasingly bioactive, bioinstructive, and patient-tailored biomaterials with greater control over the regenerative microenvironment.

### 4.6. Dental Stem Cells and Cell-Free Regenerative Strategies

Dental stem cells remain an important biological resource for regenerative dentistry because of their self-renewal, multilineage differentiation, and paracrine activity. Populations such as dental pulp stem cells (DPSCs), periodontal ligament stem cells (PDLSCs), stem cells from the apical papilla (SCAP), and stem cells from human exfoliated deciduous teeth (SHED) have been investigated for pulp–dentin, periodontal, and alveolar bone regeneration [5]. However, their therapeutic effects are increasingly understood to depend not only on engraftment and differentiation, but also on the biological signals they release into the surrounding microenvironment.

This understanding has contributed to a shift from direct cell transplantation toward cell-free regenerative strategies. Although stem-cell transplantation has produced encouraging experimental results, clinical translation is complicated by limited cell survival and engraftment, as well as the requirements for cell expansion, storage, delivery, manufacturing, and regulatory control [80,81]. Cell-free approaches seek to reproduce the paracrine effects of stem cells through products such as conditioned medium, secretomes, and extracellular vesicles (EVs). EVs are particularly relevant because they transport proteins, lipids, nucleic acids, and regulatory molecules capable of influencing recipient-cell behavior without requiring administration of viable cells [80,82].

Preclinical evidence supports the regenerative potential of these products. A systematic review of 16 studies evaluating EVs derived from oral mesenchymal stem cells reported effects associated with enhanced angiogenesis, modulation of inflammation, reduction in pro-inflammatory mediators, and improved osteogenic repair in maxillary and periodontal defects [82]. Their combination with scaffolds and hydrogels is also being investigated to improve local retention and provide more sustained delivery. This approach may be particularly valuable in periodontal and bone regeneration, where successful repair requires spatial and temporal coordination of vascularization, immune regulation, cell differentiation, and extracellular matrix remodeling [82,83].

Despite these promising findings, the translational gap remains substantial. Shanbhag et al. [83] identified 122 animal studies but only four clinical studies evaluating human-derived cell-free therapies for craniofacial regeneration. Although the limited human evidence provided initial support for safety and feasibility, it was insufficient to establish robust clinical efficacy. In addition, EV composition and biological activity can vary according to cell source, culture conditions, isolation and purification methods, dose, and delivery system, while scalable production remains an important technical challenge [81,82,83].

Overall, dental regenerative research is moving from a predominantly cell-replacement model toward a cell-instruction model, in which the signals produced by stem cells are increasingly recognized as major mediators of regeneration. Direct stem-cell transplantation remains biologically relevant, but secretome- and EV-based therapies may provide a more controllable alternative by avoiding some of the complexities associated with living-cell administration [80,81,82,83]. At present, however, their strongest evidence remains preclinical. Standardized production, characterization and dosing, optimized delivery systems, and well-designed human trials will be necessary before cell-free therapies can become predictable components of routine regenerative dental practice. A defining advance during 2020–2025 was the growing recognition of stem-cell paracrine signaling as a regenerative mechanism, which accelerated the development of secretome- and EV-based strategies as alternatives to direct cell transplantation.

### 4.7. Biomimetic Enamel Remineralization and Regeneration

Enamel regeneration is particularly challenging because mature enamel is acellular and lacks ameloblasts after eruption, leaving it without an intrinsic capacity to regenerate once tissue is lost. In addition, its mechanical properties depend on a highly organized hierarchical arrangement of hydroxyapatite crystals that is difficult to reproduce artificially [84,85]. For this reason, recent strategies have moved beyond conventional mineral replacement toward biomimetic approaches that attempt to reproduce key events of amelogenesis, including mineral nucleation, crystal growth, and structural organization.

Protein- and peptide-based systems are among the most studied approaches. Experimental models using enamel matrix protein analogs and amelogenin-derived peptides have promoted the formation of organized enamel-like hydroxyapatite and improved mechanical characteristics [84,86]. Self-assembling peptide P11-4 represents one of the most clinically advanced examples. After diffusing into early subsurface lesions, it forms a three-dimensional scaffold that promotes *de novo* hydroxyapatite nucleation and guided remineralization [87].

Clinical evidence for P11-4 is encouraging, particularly for initial carious and post-orthodontic white spot lesions. Several clinical studies summarized by Alkilzy et al. [87] reported greater lesion regression or remineralization compared with conventional fluoride-based approaches, although results vary according to the clinical outcome measured. Other emerging strategies include amorphous calcium phosphate systems, hydroxyapatite nanoparticles, dendrimers, hydrogels, and nanostructured scaffolds designed to control mineral delivery and crystal growth [86,88]. Most of these technologies, however, remain supported mainly by in vitro or preclinical evidence.

Overall, biomimetic enamel research is progressing from simple remineralization toward guided mineral formation, but true regeneration of the complete hierarchical structure of native enamel has not yet been achieved predictably. Current clinical applicability is strongest for early non-cavitated lesions, particularly with peptide-based approaches, whereas more complex amelogenesis-inspired systems remain experimental [84,85,87,89]. The main translational challenge is to demonstrate that enamel-like mineral formed under controlled conditions can reproduce the organization, mechanical behavior, and long-term stability of natural enamel in the oral environment. Progress during 2020–2025 was marked by the development of increasingly biomimetic strategies capable of guiding mineral nucleation and crystal growth, with peptide-based systems showing the clearest progression toward clinical application in early enamel lesions.

**Figure 4:**
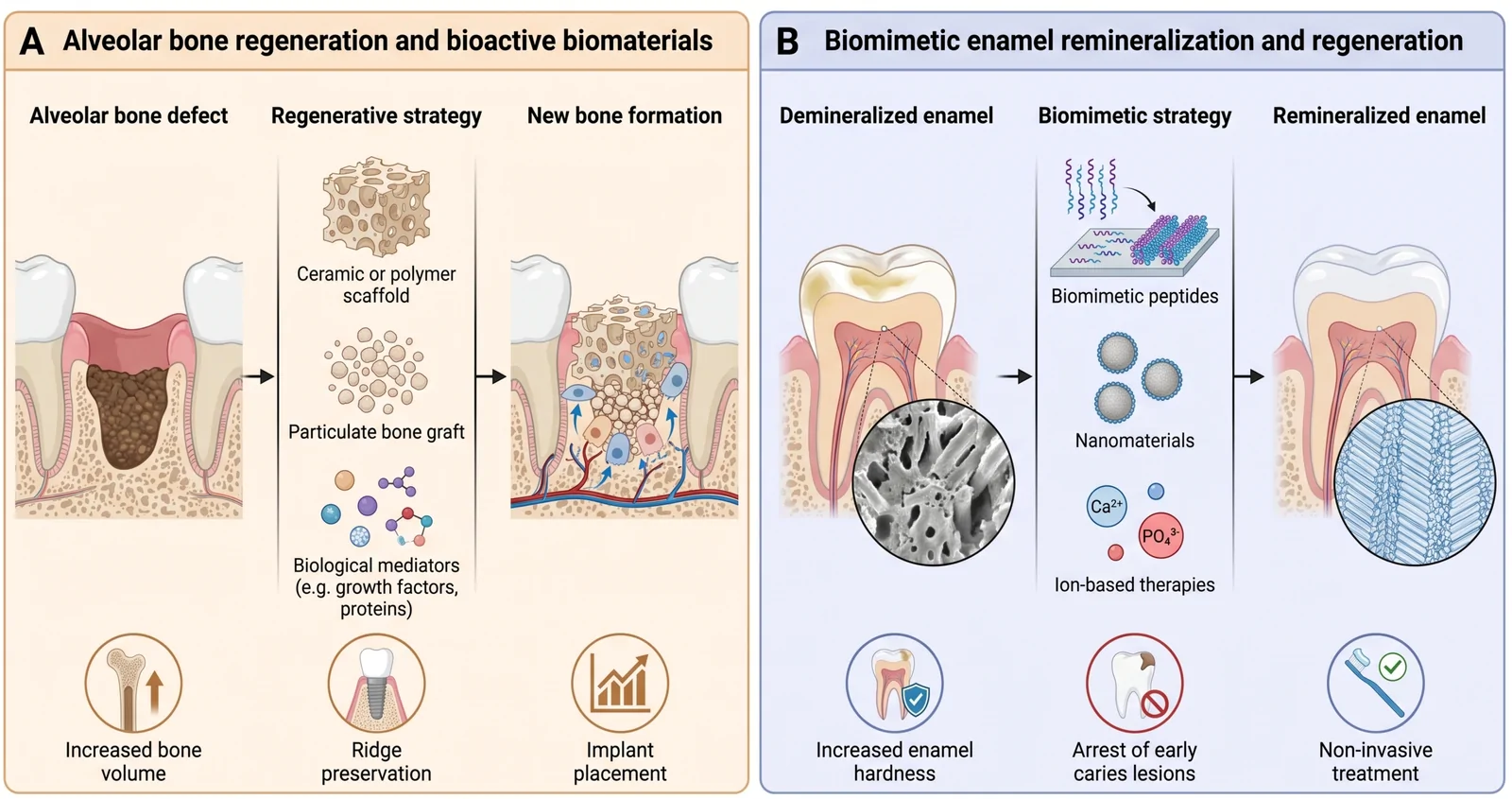
Alveolar bone and biomimetic dental tissue regeneration. Schematic representation of (**A**) alveolar bone regeneration using bone grafts, bioactive scaffolds, and biological mediators to support new bone formation, and (**B**) biomimetic enamel remineralization using different strategies (peptides, nanomaterials, and ions) to promote mineral deposition and recovery of enamel properties. The principal regenerative strategies and clinically relevant outcomes are illustrated. Created in BioRender. Valarezo, G. (2026). https://BioRender.com/zz2mf2g.

The regenerative strategies described for alveolar bone and enamel illustrate two distinct biomimetic approaches: the use of bioactive materials to support new bone formation and the use of biomimetic systems to guide mineral deposition in enamel. These strategies and their main clinically relevant outcomes are summarized in Figure 4.

### 4.8. Biofabrication, Organoids and Whole-Tooth Regeneration

Dental organoids have emerged as useful three-dimensional models for studying the cellular interactions that guide tooth development and for exploring future regenerative applications. Unlike conventional two-dimensional cultures, organoids allow cells to self-organize within an environment that better reproduces aspects of their native niche. This is particularly relevant in dental tissues, where tooth morphogenesis depends on continuous reciprocal communication between epithelial and mesenchymal compartments. Recent oral organoid models have therefore been used to investigate tooth development, epithelial stem-cell behavior, differentiation, and signaling pathways such as Wnt, TGF-β, BMP, and FGF [90,91].

One of the most relevant advances was the development of expandable epithelial organoids directly from human dental follicle tissue. Hemeryck et al. showed that these organoids retained characteristics associated with dental epithelial stemness and could undergo ameloblast differentiation. When organoid-derived epithelial cells were combined with dental pulp stem cells to form epithelial–mesenchymal assembloids, ameloblast differentiation was enhanced, with TGF-β signaling playing an important role in this response [91]. These findings provide a human experimental model for studying the epithelial–mesenchymal communication that is essential for amelogenesis and may eventually contribute to strategies aimed at reconstructing more complex dental tissues.

Tooth-germ organoids have also been investigated as an intermediate step toward whole-tooth engineering. Bektas et al. [92] combined human dental pulp stem cells and dental epithelial cells with GelMA hydrogel microparticles, which self-assembled into three-dimensional constructs while maintaining cellular viability and differentiation. The resulting structures displayed features resembling developing tooth buds, supporting the feasibility of using biomaterial-assisted self-organization to recreate some of the early events required for dental morphogenesis [92]. In parallel, advances in iPSCs, organoid culture, biomimetic scaffolds, and 3D biofabrication are providing greater control over cell distribution and tissue organization [10,93].

Whole-tooth bioengineering, however, is considerably more demanding than producing an individual dental tissue. A functional biological tooth would require coordinated formation of enamel, dentin, pulp, cementum, periodontal ligament, vasculature, and neural components, together with appropriate crown and root morphology and integration with the surrounding alveolar tissues. Whole-tooth regeneration has already been demonstrated in animal models, supporting the biological feasibility of this concept, but translation to humans remains limited [93,94]. Sui et al. [93] emphasized that although whole-tooth regeneration has progressed substantially experimentally, it remains constrained by issues related to functional integration, immune responses, long-term stability, and control of tissue development.

From a translational perspective, dental organoids and whole-tooth bioengineering are therefore at very different stages from clinically established regenerative procedures. Organoids currently provide valuable platforms for developmental modeling, disease research, and identification of regenerative cell populations, while their direct therapeutic use remains experimental [10,90,91]. Whole-tooth bioengineering is even further from routine clinical application because reliable human epithelial cell sources, predictable tooth morphology, vascularization, innervation, periodontal attachment, and long-term functional integration still need to be achieved [10,93,94]. Thus, the major advance between 2020 and 2025 has been the increasing ability to reproduce and manipulate key developmental processes in three-dimensional systems, rather than the clinical availability of a bioengineered replacement tooth. This progress was reflected particularly in the development of human dental organoids, epithelial–mesenchymal assembloids, and biomaterial-assisted tooth-germ models that more closely reproduce the cellular organization and signaling involved in early tooth development.

**Figure 5:**
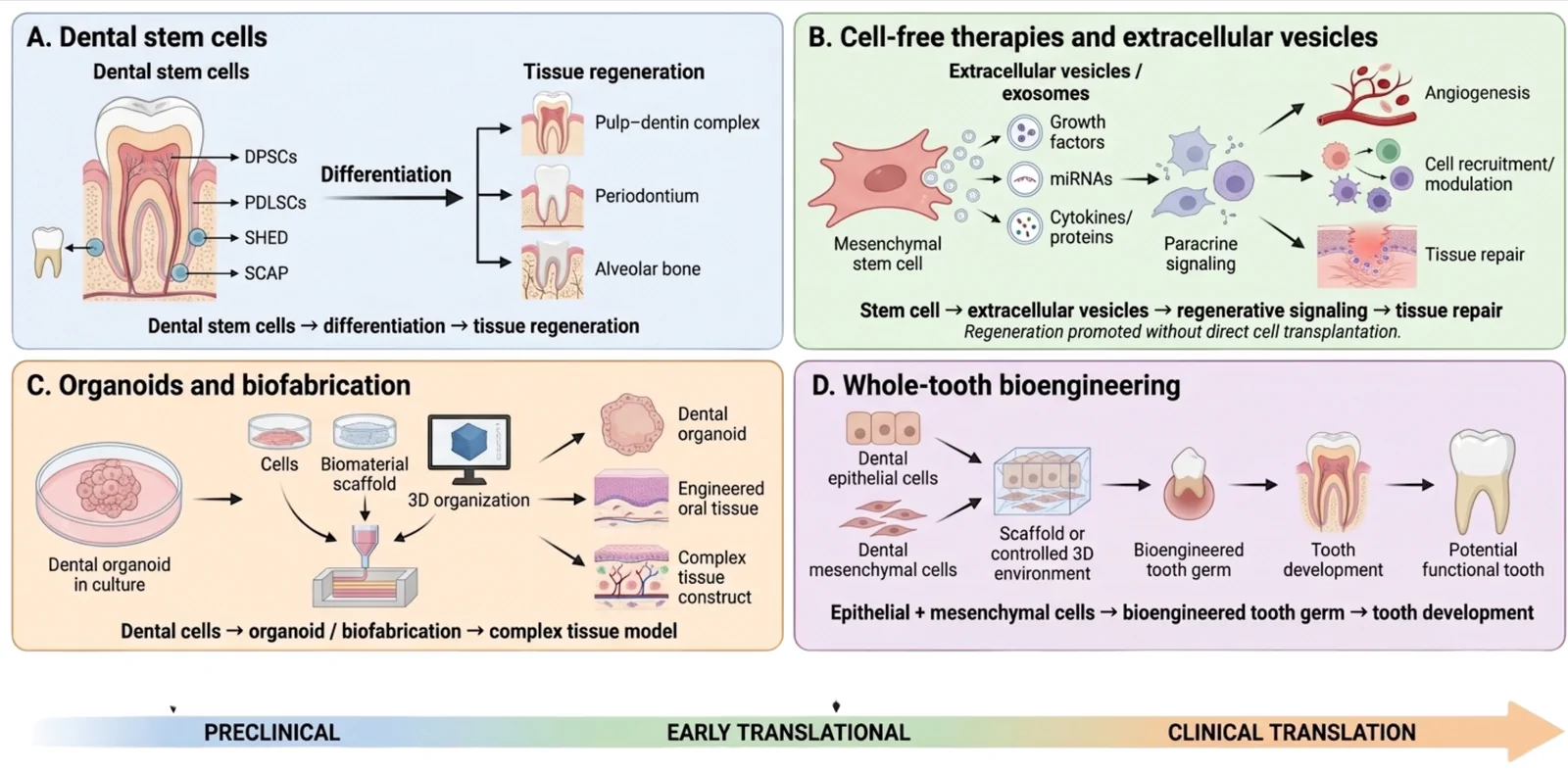
Emerging and future regenerative strategies in dentistry. Schematic representation of (**A**) dental stem cell-based approaches, (**B**) cell-free therapies using extracellular vesicles, (**C**) organoids and biofabrication, and (**D**) whole-tooth bioengineering. These strategies represent different levels of biological complexity and translational maturity, ranging predominantly from preclinical research to early translational development. Created in BioRender. Valarezo, G. (2026). https://BioRender.com/hzfe0pv.

These emerging approaches illustrate the progressive transition from cell-based and cell-free strategies to increasingly complex three-dimensional regenerative systems. Dental stem cells, extracellular vesicles, organoids, biofabrication, and whole-tooth engineering are currently at different stages of development, with most advanced strategies remaining predominantly preclinical or at early translational stages, as summarized in Figure 5.

The major regenerative strategies discussed above differ considerably in their level of biological complexity, supporting evidence, and clinical translation. Table 1 summarizes the main advances, current evidence, translational status, and remaining limitations across the eight regenerative areas reviewed.

## 5. Challenges and Limitations

Despite the progress achieved in regenerative dentistry, an important limitation across the field is the difference between obtaining a favorable clinical outcome and restoring the original structure and function of a tissue. In regenerative endodontics, periapical healing or continued root development does not necessarily indicate reconstruction of a native pulp–dentin complex [43,50]. Similarly, enamel remineralization is not equivalent to complete enamel regeneration [84,87,88], while periodontal regeneration requires coordinated restoration of cementum, periodontal ligament, and supporting bone rather than isolated tissue formation [52,53,57]. These distinctions are consistent with the evidence discussed within the respective regenerative axes.

A second challenge is the biological complexity of oral tissues. Successful regeneration often requires cell survival and differentiation to occur together with vascularization, immune regulation, extracellular matrix remodeling, and spatial organization. These requirements become increasingly difficult in complex structures such as the pulp–dentin complex [47,48], periodontal attachment apparatus [57,58,59], and especially the whole tooth, where several mineralized and soft tissues must develop and integrate in a coordinated manner [10,90,91,92,93]. For this reason, favorable findings in isolated tissues or experimental models cannot automatically be interpreted as functional regeneration. The whole-tooth literature in your manuscript specifically emphasizes the need for coordinated formation and integration of multiple dental tissues and the predominantly experimental status of these approaches.

Reproducibility also remains a major concern. Differences in cell sources, biomaterial composition, scaffold design, preparation protocols, and delivery systems can substantially influence treatment outcomes. This is particularly evident with platelet concentrates, whose final characteristics depend strongly on preparation conditions [69,71,72], and extracellular vesicles, whose composition and biological activity may vary according to cell source, culture conditions, isolation method, dose, and delivery system [80,81,82,83]. Advanced biomaterials face similar difficulties because mechanical stability, degradation, porosity, and bioactivity must be balanced according to the target tissue [63,64,65,66,67,68,75,76,77,78]. Greater standardization is therefore needed before results can be compared reliably across studies and translated into routine practice.

Finally, a substantial gap remains between preclinical evidence and human application. Several advanced strategies, including engineered periodontal constructs [57,58,59,60,61,62], cell-free therapies [80,81,82,83], next-generation membranes [63,64,65,66,67,68], and organoid and whole-tooth bioengineering approaches [10,90,91,92,93], remain supported mainly by in vitro, animal, or early translational evidence. Clinical translation also introduces additional challenges related to manufacturing, scalability, quality control, long-term safety, and regulatory requirements. Future progress will therefore depend not only on improving biological performance, but also on developing reproducible approaches supported by standardized protocols and well-designed long-term clinical studies [78,81,83,90,93].

## 6. Future Perspectives in Regenerative Dentistry

Future progress in regenerative dentistry will likely depend on moving from approaches that stimulate isolated biological responses toward strategies capable of controlling the regenerative microenvironment as a whole. Rather than promoting cell proliferation, mineralization, or tissue formation independently, emerging therapies increasingly seek to coordinate cell behavior, immune modulation, angiogenesis, extracellular matrix remodeling, and tissue-specific differentiation. This transition is particularly relevant for complex structures such as the pulp–dentin complex and periodontal attachment apparatus, where functional regeneration requires the organized integration of several cellular and tissue components [47,48,57,58,59,80,81,82,83].

The integration of complementary technologies may further support this transition. Advanced biomaterials can provide spatial and mechanical support while also serving as delivery systems for bioactive signals, including extracellular vesicles [76,78,81,82,83]. At the same time, three-dimensional fabrication, organoids, and tooth-germ models are improving the ability to reproduce developmental environments and control cellular and tissue organization [10,90,91,92,93]. These developments suggest that future regenerative therapies may increasingly combine biological signals with engineered microenvironments rather than relying on a single cell type, biomaterial, or regenerative stimulus.

However, the clinical impact of these advances will ultimately depend on stronger human evidence. Future studies should prioritize standardized protocols, clinically meaningful outcomes, adequate follow-up, and direct comparisons with established treatments. Particular attention should be given to demonstrating not only tissue formation but also stable structural and functional recovery. Bridging this translational gap will be essential for determining which emerging technologies can progress from promising experimental strategies to predictable regenerative treatments in routine dental practice [62,68,78,83,90,93].

## 7. Conclusions

Regenerative dentistry has progressed from approaches primarily focused on tissue replacement toward biologically driven strategies aimed at restoring tissue structure and function. Advances between 2020 and 2025 have strengthened established approaches such as regenerative endodontics, periodontal regeneration, GTR/GBR, platelet concentrates, and alveolar bone regeneration, while also expanding the possibilities offered by bioactive biomaterials, dental stem cells, cell-free therapies, biomimetic enamel regeneration, organoids, and whole-tooth bioengineering. However, these strategies remain at markedly different stages of development, ranging from procedures supported by human evidence and already incorporated into clinical practice to technologies that are still predominantly experimental.

A central finding of this review is that successful healing or new tissue formation should not automatically be interpreted as complete biological regeneration. Reconstructing the organization and function of native oral tissues remains particularly challenging when vascularization, innervation, multiple tissue interfaces, and precise spatial organization are required. Future progress will therefore depend on narrowing the gap between promising preclinical findings and predictable clinical outcomes through greater standardization, stronger long-term human evidence, and better control of the regenerative microenvironment. Ultimately, the clinical value of emerging regenerative technologies will depend not only on their ability to generate new tissue, but also on their capacity to achieve safe, stable, and functional regeneration that provides a meaningful advantage over established treatments.

Looking ahead, regenerative dentistry may become part of a broader model of oral care that combines tissue restoration with health promotion, disease prevention, early detection, and long-term follow-up. Within this context, artificial intelligence and other digital health technologies may complement regenerative approaches by supporting risk assessment, early disease detection, personalized treatment planning, clinical decision-making, and monitoring of therapeutic outcomes. Digital and interactive educational tools may also contribute to patient education, preventive behaviors, and sustained engagement in oral health care. Integrating these preventive, regenerative, and digital perspectives could support a more personalized and patient-centered approach to dentistry, in which preserving oral health and preventing further tissue damage remain as important as developing increasingly sophisticated strategies for tissue regeneration.

## Figures and Tables

**Table 1:** Translational overview of major advances in regenerative dentistry reported between 2020 and 2025.

Regenerative Area	Main Regenerative Strategy	Current Evidence	Main Advances	Major Limitations	Translational Status	Key References
Regenerative endodontics and pulp–dentin regeneration	Cell homing, REPs, DPSCs, biomimetic scaffolds and signaling modulation	Clinical evidence for cell-free REPs; cell-based approaches remain less developed	Continued root development and biological repair in immature teeth	True pulp–dentin regeneration, vascularization, innervation and tissue organization remain challenging	**Clinical/early translational**	[43,44,45,46,47,48,49,50,51]
Periodontal regeneration and cementum–PDL engineering	PDLSCs, multiphasic scaffolds, cell sheets and signaling modulation	Clinical evidence for conventional approaches; advanced engineering mainly preclinical	Reconstruction increasingly targets the cementum–PDL–bone complex	Functional fiber insertion and predictable tissue integration remain difficult	**Clinical/preclinical**	[52,53,54,55,56,57,58,59,60,61,62]
GTR/GBR	Barrier membranes and advanced bioactive or functionally graded systems	Strong clinical basis for conventional membranes	Evolution from passive barriers toward bioactive regenerative interfaces	Mechanical stability, degradation and clinical validation of advanced membranes	**Established clinical/early translational**	[63,64,65,66,67,68]
Platelet concentrates	PRP, PRF and L-PRF	Clinical studies, RCTs and systematic reviews	Autologous fibrin matrices may enhance early healing	Heterogeneous protocols and inconsistent evidence of long-term superiority	**Clinical adjunct**	[69,70,71,72,73,74]
Alveolar bone regeneration	Synthetic substitutes, DDM and bioactive/bioinstructive biomaterials	Clinical evidence for several conventional materials; advanced systems mainly preclinical	Transition toward biomaterials that actively influence regeneration	No material fully reproduces autologous bone; advanced systems require greater clinical validation	**Clinical/translational**	[75,76,77,78,79]
Stem cells from the apical papilla (SCAP) and cell-free strategies	Dental MSCs, secretome and EVs	Strong preclinical evidence; limited human evidence for cell-free therapies	Shift from cell replacement toward paracrine and cell-instruction approaches	Standardization, production, dosing and limited clinical validation	**Preclinical/early clinical**	[5,80,81,82,83]
Biomimetic enamel regeneration	Peptides, ACP and nanostructured mineralization systems	Human evidence for selected peptide approaches; most systems remain experimental	Controlled mineral nucleation and organization increasingly mimic amelogenesis	Complete structural regeneration of native enamel remains unresolved	**Emerging clinical/preclinical**	[84,85,86,87,88,89]
Organoids and whole-tooth bioengineering	Dental organoids, assembloids, tooth-germ constructs and biofabrication	Primarily in vitro and animal evidence	Increasing ability to reproduce epithelial–mesenchymal interactions and early odontogenesis	Morphology, vascularization, innervation, enamel formation and functional integration	**Experimental/preclinical**	[10,90,91,92,93,94]

Abbreviations: REP, regenerative endodontic procedure; DPSC, dental pulp stem cell; PDL, periodontal ligament; PDLSC, periodontal ligament stem cell; GTR, guided tissue regeneration; GBR, guided bone regeneration; PRP, platelet-rich plasma; PRF, platelet-rich fibrin; L-PRF, leukocyte- and platelet-rich fibrin; DDM, demineralized dentin matrix; SCAP, stem cells from the apical papilla; SHED, stem cells from human exfoliated deciduous teeth; EV, extracellular vesicle; ACP, amorphous calcium phosphate.

## Data Availability

No new data were created or analyzed in this study. Data sharing is not applicable to this article.
